# Mobile application tool for remote rehabilitation after discharge from coronavirus disease‐19 rehabilitation unit

**DOI:** 10.1049/htl2.12033

**Published:** 2022-08-08

**Authors:** Daniele Emedoli, Federica Alemanno, Elise Houdayer, Luigia Brugliera, Sandro Iannaccone, Andrea Tettamanti

**Affiliations:** ^1^ Department of Rehabilitation and Functional Recovery IRCCS Ospedale San Raffaele Milan Italy

## Abstract

A smartphone application (Medico‐Amico) has been developed by the collaboration of San Raffaele Scientific Institute and Khymeia Group S.R.L. with the aim of providing physical exercises and communicating with patients after their hospitalization in a coronavirus disease (COVID)‐rehabilitation unit. Thirty patients used the application for remote rehabilitation for 4 weeks. They were prescribed personalized motor exercises to perform three times a week. Clinicians could interact with each patient by an encrypted video call in order to give encouragement, mental support, modify intensity during training sessions, or to prescribe new exercises. Patients were asked to perform motor exercises and also to monitor their vital signs, such as temperature, blood pressure, and oxygen saturation, inserting scores in a specific section of the application. After 4 weeks of remote rehabilitation patients showed improvements in independence during activity of daily living and strength. Also, satisfaction and mobile application usability scores reached patients’ appreciation and enjoyment.

## 1 INTRODUCTION

Coronavirus disease 2019 (COVID‐19) is a global pandemic caused by the severe acute respiratory syndrome coronavirus‐2 (SARSCoV‐2) [1, 2, 3]. COVID‐19 can lead to the occurrence of a variety of symptoms such as fever, cough, increased airway secretions, dyspnea etc. Furthermore, patients might incur weakness, sarcopenia and decreased exercise tolerance due to long‐term bed rest [4]. Previous studies demonstrated that more than 20% of sub‐intensive and 50% of intensive care patients required respiratory, motor or cognitive rehabilitation after the acute stage of the disease [5, 6, 7, 8, 9, 10, 11]. To provide patients with adequate rehabilitation following the acute phase of the disease, many hospitals created dedicated COVID‐19 rehabilitation departments, since approximately 30% of hospitalized COVID‐19 patients needed rehabilitation for functional impairments [7]. Safe rehabilitation of COVID‐19 patients might represent an issue for the medical staff. Such risks have been addressed by the World Confederation for Physical Therapy and other authorities including the Association of Physical Therapy, and guidelines have been published to recommend methods of respiratory rehabilitation and physical therapy for COVID‐19 patients in all stages of the disease [4].

After hospital discharge, post COVID‐19 patients might have the need to continue functional rehabilitation. However, due to restrictions and transportation difficulties, rehabilitation might be a challenge for patients. As a consequence, many healthcare institutions, including the San Raffaele Scientific Institute (Milan, Italy), implemented COVID‐19 telerehabilitation and remote rehabilitation units, with the aim of improving patients’ functioning, self‐care behaviour, to monitor patients’ recovery and achieve better health‐related outcomes [12].

Many attempts have been made to improve self‐management skills and health‐related outcomes using information technology such as telephone calls, video conferencing and the Internet [13]. San Raffaele Scientific Institute, in collaboration with Khymeia Group, developed a smartphone application (Medico‐Amico) for clinicians providing exercises and communication with patients, after hospital discharge, to improve their recovery and monitor symptoms over time.

The application gave clinicians the possibility to create personalized physical exercises, remotely customizable on patients’ needs, assign exercises prescription over a period of time, remotely monitor patients’ vital signs, video‐call or chat with patients in real time and assess the execution of the exercises.

The primary aim of this study was to investigate whether this novel, low‐cost and environmentally friendly method of remote rehabilitation training and communication was feasible and acceptable for patients after being discharged from COVID‐19 rehabilitation departments. Secondarily, we aimed at collecting clinical and adherence data to better define the efficiency of such intervention.

## 2 MATERIAL AND METHODS

### 2.1 Patients’ population

Thirty post‐COVID‐19 patients discharged from the COVID‐19 Rehabilitation Department of the San Raffaele Scientific Institute (Milan, Italy) between April 2020 and June 2021 were recruited and considered eligible for this single arm, monocentric, clinical study. Further inclusions criteria were age between 18 and 75 years, willingness and ability to perform therapeutic exercises following visual and verbal instructions as well as sufficient knowledge of the Italian language. Exclusion criteria were any physiological or mental disease which could impair the ability to perform therapeutic exercises, already planned physical therapy during the intervention period, inability to operate a smartphone (e.g. vision, hearing, cognitive or dexterity impairment), absence of an internet connection at home and pregnancy. The presence of a caregiver was also recommended.

In order to include participants regardless of their socio‐economic status, to those without the possession of a smartphone (Android, iOS or web browser), the Department of Rehabilitation and Functional Recovery (San Raffaele Scientific Institute) provided them one.

### 2.2 Assessments and patients’ flow

After obtaining written consent, patients were clinically evaluated and then instructed to download and use the mobile application. After the first training session, personalized therapeutic exercises were identified and set up on the application by specialized physiotherapists. Patients were encouraged to perform the exercises by themselves, 3 days a week for 4 consecutive weeks.

Patients were assessed after hospital discharge (T0) and after the 4‐week remote rehabilitation program (W4) with the following clinical measurement scales: activity of daily living (functional independence measure [FIM]), lower limb functioning (short physical performance battery [SPPB]), balance and fall risk (Berg Balance Scale [BBS]), mobility and walking ability (timed up and go test [TUG]) and strength (30 second sit to stand test).

In addition, at W4, patients’ satisfaction, adherence to program and application usability were also assessed. Patients’ satisfaction (Client Satisfaction Questionnaire), and application usability (System Usability Scale) were evaluated using specific questionnaires. When needed, semi‐structured interviews following standard guidelines for qualitative research [14] were used. Adherence to the program was measured using the automatic percentage of exercise completion provided by the application.

**FIGURE 1:**
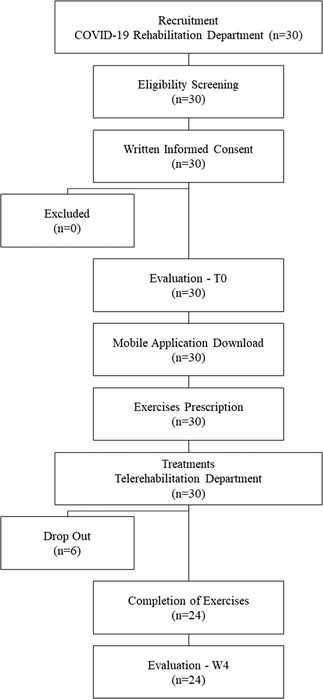
Patient flow chart

Patients’ flow chart and drop‐outs are reported in Figure 1.

### 2.3 Outcome measurements

FIM: TheFIM is an 18‐item, seven‐level, ordinal scale intended to be sensitive to changes over the course of a comprehensive inpatient medical rehabilitation program. It includes measures of independence for self‐care, including sphincter control, transfers, locomotion, communication and social cognition [15]. The tool is used to assess a patient's level of disability as well as a change in patient's status in response to rehabilitation or medical intervention [16, 17].

SPPB: The SPPB is a group of measures that combines the results of the gait speed, chair stand and balance tests [18]. It has been used as a predictive tool for possible disability and can aid in the monitoring of function in older people. The scores range from 0 (worst performance) to 12 (best performance).

BBS: The BBS is a 14‐item measure that assesses balance in static and dynamic conditions in adults. The BBS consists in 14 items scored on a 5‐point ordinal scale, ranging from 0 to 4 (0 indicating lowest level of function; 4 indicating highest level of function), with a maximum total score of 56. Participants presenting a score between 41 and 56 points have been described as ‘independent’; scores between 21 and 40 are interpreted as ‘walking with assistance’; and scores between 0 and 20 are generally classified as ‘wheelchair bound’ [19].

TUG: The TUG assesses mobility, balance, walking ability and fall risk in older adults. Previous research suggests that the TUG test has some predictive ability in community dwelling people (predicting ability to walk outside alone) and it is suggested that it may have some capacity to predict function in other settings [20].

30 Second Sit to Stand Test: The 30‐second Chair‐Stand Test (30s‐CST) is a single‐item physical performance tool for the assessment of lower body strength. It is performed by counting the number of stands completed in 30 s with hands crossed against the chest [21]. The simplicity of the test makes it easy to use, requiring less than 5 min.

Client Satisfaction Questionnaire: The Client Satisfaction Questionnaire (CSQ‐8) [22] is a multi‐item measure that has been used frequently in research and health care literature [23, 24]. CSQ‐8 is a short 8‐item tool that has a good reliability and validity when used across a range of settings [25, 26]. CSQ‐8 is a structured survey used to assess the level of satisfaction of care. Items are scored on a Likert scale from 1 (low satisfaction) to 4 (high satisfaction) with different descriptors for each response point. The total score ranges from 8 to 32, with higher scores indicating greater satisfaction.

System Usability Scale: The System Usability Scale (SUS) provides a quick, reliable tool for measuring the usability of a system. It consists in a 10‐item questionnaire with five response options for respondents, ranging from ‘Strongly agree’ to ‘Strongly disagree’. [27].

Adherence Diary: Adherence Diary were filled automatically by Medico‐Amico during the Remote Rehabilitation sessions. It consists in a digital diary that assesses percentage of exercises execution. The system also records each repetition, scores and time duration during each session in order to provide an automated diary of performances expressed in percentage of completion.

### 2.4 Mobile application development

The smartphone application was developed by the collaboration of San Raffaele Scientific Institute and Khymeia Group, with the aim of providing patients with exercise videos in association with a communication tool between them and their clinicians. Medico‐Amico was approved as a class 1 medical device by the Italian Ministry of Health and registered in the Italian Medical Device Registry (93/42/EEC; IEC 60601‐1:2007). The utilized version contained the videos of an animated avatar performing 33 therapeutic exercises divided into four different macro‐areas of intervention: general mobility exercises, strength exercises, respiratory accessory muscles training and walking exercises (see [Link], [Link]). Each exercise included an introductive video with verbal instructions and one video to go along while exercising. Patients could perform exercises following the repetitions scheme that was reproduced by the application. For each exercise, clinicians could set up the execution speed, execution time and number of repetitions.

The FrontEnd of the application was developed on the IONIC platform, which allowed deployment on iOS, Android and as a web application usable from any web browser on any operating system. The BackEnd of the application was developed on the NodeJS platform, and is resident on a CentOS virtual machine and has been implemented according to PaaS (Platform as a Service) logic on the Amazon Web Services architecture. The application Database is present only on the BackEnd. Altas MongoDB Database was chosen as the Database platform, with limited access to the web application only.

### 2.5 Users and access

The application had three types of users: structure, doctor (clinicians) and patient. The three users were hierarchically structured: only structure accounts could invite doctors, and only a doctor account could invite patients. Patients could download independently the application from the stores, but without having approval by a Doctor, they could not carry out any activity.

### 2.6 Operations

The following activities could undergo between doctors and patients:

- persistent chat (maintained over time), asynchronous;
- audio‐video conference (which can only be started by a doctor);
- starting exercises in real time: during an audio/video call, the doctor can select and launch one or more exercises on the patient's device in real time.

All operations take place exclusively on encrypted modalities, managed by BackEnd software.

### 2.7 Prescriptions

Patients had access to their own calendar, and could simply start the prescribed activity. An activity prescribed on a given day could be performed at any time, regardless of the time defined by the doctor for that activity.

Doctors could visualize, on the calendar of a specific Patient, which prescribed activities have been carried out and the specific results.

### 2.8 Data protection

The privacy policy is provided in the app. When creating an account (which is configured to all intents and purposes as a data controller in the context of the GDPR), the data controller provides Khymeia Group with a specific text of privacy policy that is available to all users (structure, doctor, patient) referring to that specific user. The privacy notice text was prepared following the recommendations of art 12, 13, 14 GDPR.

During the study, the collection, storage and management (including subsequent withdrawal) of consents to the processing of personal data by users was managed outside the platform, with modalities and methods defined by the data controller.

### 2.9 Interventions

After recruitment and eligibility screening, patients signed an informed consent and performed a screening evaluation (T0) in which were evaluated clinical outcomes such as activity of daily living, lower limb functioning, balance, fall risk, mobility, walking ability and strength. After this, each patient downloaded Medico Amico from Google Play store or Apple store (depending on mobile device) with assistance of caregiver or clinicians. Then, physiotherapists personalized a setting of exercises, tailored on the estimation of the individual patients’ needs. After it, a specialized physiotherapist from telerehabilitation department took charge of patients contacting them and starting the prescription of exercises. Moreover, the clinicians from telerehabilitation department could contact each patient once a week to give encouragement, mental support, modify intensity during training sessions or to prescribe new exercises.

Also, clinicians could interact with patients during exercises by an encrypted video call in order to check the execution of exercises and suggest improvements during training. It was possible to customize the therapeutic intervention by monitoring the execution of the exercises during video call and assessing the percentage of exercises execution, tailoring treatments on capacity and real execution of exercises. If some exercises were not completed by patients, clinicians could contact them and ask reasons why they were not completed, encouraging then when necessary, or adapting the exercises.

Participants were also asked to monitor their vital signs, such as temperature, blood pressure and oxygen saturation, and to insert scores in a specific section of the app. Physicians regularly checked vital signs of patients and, when needed, could also prescribe medications via app.

### 2.10 Statistical analysis

Based on previous evidence [28], a power analysis was carried out assuming that the total FIM (primary outcome) after remote rehabilitation training would increase 3.8 points (with a standard deviation of 5.61). Considering a drop‐out rate of 20%, 30 participants would be needed for 90% power to detect significant within group improvements (α = 0.05) after intervention. Data distribution was assessed using the Shapiro–Wilk test and non‐parametric tests were used to analyse data. Longitudinal changes (T0‐W4) were assessed using linear mixed‐effects models. Such models were adjusted for the baseline value of each considered variable. *P* values were Bonferroni‐corrected for multiple comparisons at *P *< 0.05. All statistical analyses were performed using RStudio statistical software (version 1.4.1717; 2009–2021 RStudio, PBC). Descriptive analyses of W4 adherence, usability and satisfaction were performed.

## 3 RESULTS

A total of 30 patients (mean age 60.3 ± 10.8 years; 8 female; BMI 25.3 ± 2.9) were included in this study. Out of the 30 patients, 24 participants completed the full study procedure and measurements. Six patients dropped out of our study because they never used the mobile application (four patients) or started other rehabilitation programs (two patients). All data collected from the remaining 24 patients have been analysed.

**FIGURE 2:**
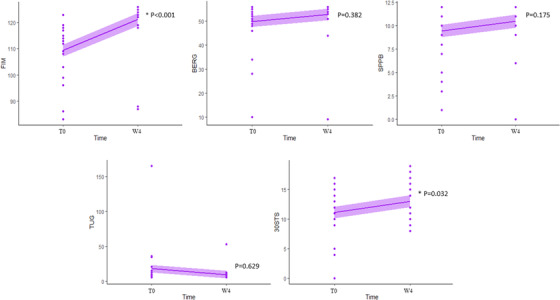
Clinical outcomes. Activity of daily living (FIM, functional independence measure), balance and fall risk (BBS, Berg Balance Scale) lower limb functioning (SPPB, short physical performance battery), mobility and walking ability (TUG, timed up and go test), and strength (30s‐CTS, 30 second sit to stand test). Significance * (*P* ≤ 0.05)

After 4 weeks of remote rehabilitation, post‐COVID‐19 patients showed statistically significant improvements in the clinical outcomes FIM [12.00 (7.00; 14.50) *P *< 0.001] and 30s‐CST [01.00 (−00.50; 05.00) *P *= 0.030] (Figure 2). Conversely, BBS, SPPB and TUG did not show significant differences before and after training (Table 1).

**TABLE 1:** Clinical outcomes

Outcome		Mean (SD)	Median (1st–3rd quartiles)	P* T0–W4
FIM	T0	108.08 (11.03)	112.00 (106.75; 114.00)	* < 0.001
	W4	120.75 (10.59)	124.50 (121.25; 126.00)	
	Δ *delta*	12.67 (10.87)	12.00 (7.00; 14.50)	
BBS	T0	49.42 (11.60)	54.00 (50.00; 56.00)	0.382
	W4	52.52 (10.31)	56.00 (54.00; 56.00)	
	Δ *delta*	3.10 (7.48)	00.00 (00.00; 02.00)	
SPPB	T0	9.30 (3.55)	11.00 (07.50; 12.00)	0.175
	W4	10.52 (2.73)	12.00 (10.00; 12.00)	
	Δ *delta*	1.21 (2.78)	00.00 (00.00; 02.00)	
TUG	T0	19.57 (34.40)	09.00 (08.00; 15.00)	0.629
	W4	9.87 (10.07)	07.30 (06.00; 08.00)	
	Δ *delta*	−9.7 (34.28)	−02.00 (−04.00; 00.00)	
30s‐CST	T0	11.26 (4.28)	12.00 (10.00; 14.00)	* 0.030
	W4	13.37 (3.44)	14.00 (10.50; 15.50)	
	Δ *delta*	2.10 (3.80)	01.00 (−00.50; 05.00)	

Activity of daily living (FIM, Functional Independence Measure), balance and fall risk (BBS, Berg Balance Scale) lower limb functioning (SPPB, Short Physical Performance Battery), mobility and walking ability (TUG, timed up and go test) and strength (30s‐CTS, 30 Second Sit to Stand Test). SD, standard deviation. Significance * (*P*≤0.05).

Descriptive analyses of adherence, usability and satisfaction showed high percentages of adherence (74.4 ± 40.8%), SUS (88.7 ± 13.7/100%) and CSQ‐8 (84.3 ± 16.8%) (Table 2).

**TABLE 2:** Mobile application outcomes

Outcome	Mean (SD) %
ADHERENCE	74.4 (40.8) %
SUS	88.7 (13.7) %
CSQ‐8	84.3 (16.8) %

Adherence (percentage of adherence), mobile application usability (SUS, System Usability Scale), patients’ satisfaction (CSQ‐8, Client Satisfaction Questionnaire). SD, standard deviation.

## 4 DISCUSSION

The development of a mobile application for telerehabilitation by the San Raffaele Scientific Institute with the collaboration of Khymeia Group S.R.L has been a prominent contribution for the management of patients discharged from our COVID‐19 Rehabilitation Unit.

Adherence to motor exercises program during remote rehabilitation reached scores of 74.4 ± 40.8%. This high adherence to the rehabilitative program seemed to optimize patients’ recovery, which was particularly highlighted by the FIM and 30s‐CST results. We particularly expected improvements in these two physical domains since most of the prescriptions had the aim to ameliorate activities of daily living and strength more than balance, mobility and walking abilities for which patients did not show signs of dependence at hospital discharge. Moreover, these latter domains (balance, mobility and walking) are even now demanding to train during telerehabilitation because these would require close supervision during exercises execution.

Pandemic‐related lockdown, transport limitation and cost of traditional physiotherapy constitute obstacles to the continuity of care for both patients and health care providers after patients’ discharge from hospitals. The development of electronic devices for the management of health, including application for smartphones, has been on the rise in the last decade. Many smartphone's applications providing exercises for patients are now available [29, 30, 31]. Generally, those applications present with screening questions at patient's entry, and provide a limited specificity of exercises’ prescription. Exercises are not tailor‐made or adapted to patients’ specific needs. Moreover, following an initial consultation with a practitioner, patients are left training by themselves and are not provided with any interaction with clinicians or therapists during the training program.

The Medico‐Amico application that we developed and tested in this study presented a totally different approach, creating a communication interface between patients and clinicians and providing specific exercises for rehabilitation of patients discharged after COVID‐19 infection. The results of our semi‐structured interviews after training demonstrated that this smartphone application was feasible and acceptable for patients, with a high percentage of satisfaction and usability. Importantly, our research team provided patients with ongoing support and consistent reinforcement using the smartphone application. Patients were actively engaged in self‐care behaviour and provided with timely feedback regarding their symptoms, exercises’ execution, including advice on behavioural changes. Individualized goals for exercise, careful monitoring of each participant, providing feedbacks using convenient technology, all might have influenced patients’ behavioural change. These positive findings regarding patients’ self‐care behaviour were supported by the fact that adherence reached high scores.

It is noteworthy that, despite our positive findings, this study presented some limitations. First, we did not measure clinical outcomes at 3 months’ follow‐up, which may have given better understandings of usability and satisfaction of our remote rehabilitation program. Second, due to the pandemic, no control group could have been included in the study. Thus, studies taking into account these limitations should be run to further investigate the benefits of such technology on patients’ recovery and health.

## 5 CONCLUSION

Continuous efforts are needed to motivate patients to be more active after discharge from Rehabilitation Departments since an active lifestyle has been shown to have significant relationships with better health outcomes. Smartphone applications have the potential to address the complexity of non‐adherence behaviours regarding lifestyle modifications. In this study, we showed that the use of a smartphone‐based interactive tool improved patients’ adherence to a training program, and that this adherence could be associated with a trend towards improved activity and lifestyle changes. In addition, the application seemed to optimize usability and treatment satisfaction in post COVID‐19 patients.

Further studies with larger sample sizes and control group are needed to show possible impact on clinically relevant outcomes.

## CONFLICT OF INTEREST

D. Emedoli received compensation for consulting services, speaking activities and clinical development from Khymeia Group S.R.L. The other authors declared no potential conflicts of interest with respect to the research, authorship and/or publication of this article.

## FUNDING INFORMATION

The author(s) received no specific funding for this work.

## Supporting information

Supporting Information

Supporting Information

## Data Availability

Data available on request from the authors. The data that support the findings of this study are available from the corresponding author upon reasonable request.
