# Supplementary material for: Mobile application tool for remote rehabilitation after discharge from coronavirus disease‐19 rehabilitation unit
Source: Healthc Technol Lett. 2022 Aug 8;9(4-5):70–6. doi: 10.1049/htl2.12033 (PMC9535743; doi:10.1049/htl2.12033)
Supplement: Supplementary file 1 — Supporting Information [file HTL2-9-70-s002.docx]

| **Name Exercise** | **Primary Instruction** | **Compensation Instruction** | **Figure** |
| --- | --- | --- | --- |
| Humerus Flexion 0-180 | Lie on your back, put your hands together and bring your arms up as shown in the video | Keep your back straight | 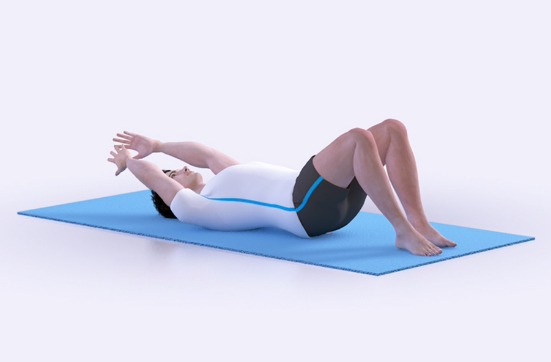 |
| Hip Flexion (alternate) | Lie on your back, flex your hips alternately, adding a little over-thrust with your arms as shown in the video | Alternate legs | 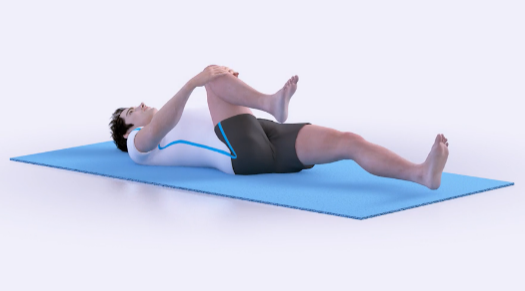 |
| Trunk Rotation 1 | Lie on your back, keeping your knees flexed, slowly rotate your legs to one side and the other as shown in the video |  | 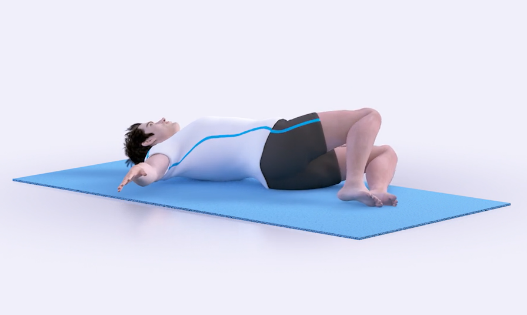 |
| Scapular Retraction | While sitting, bring your hands to your hips and try to bring your elbows closer to each other by widening your chest as shown in the video | Leave your hands anchored on your hips | 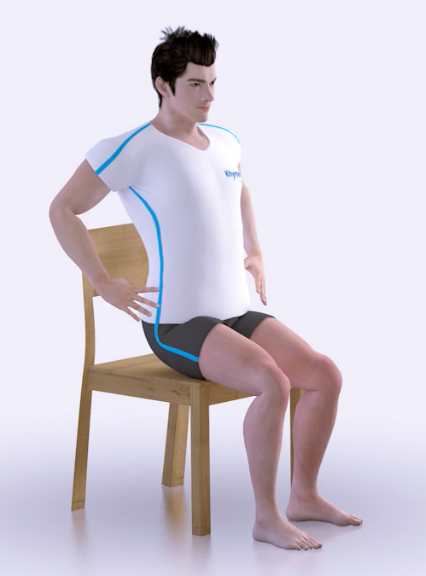 |
| Scapular-humeral Mobility | While sitting, try to bring your hands together along your back trying to bring them together as shown in the video | Always return to a neutral position with your hands on your thighs to exhale; keep your back straight | 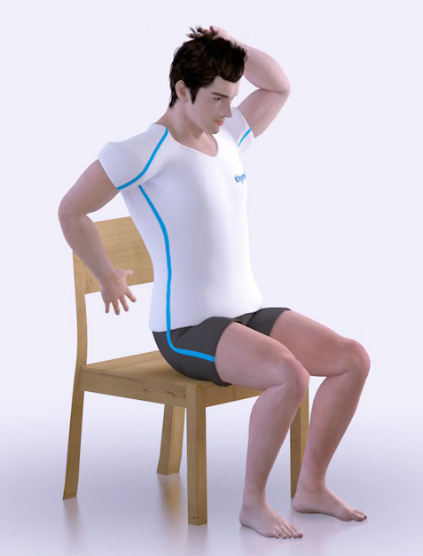 |
| Clockwise Scapular Circling | While seated, rotate your shoulder blades to make large, slow circles clockwise as shown in the video | Keep your back straight | 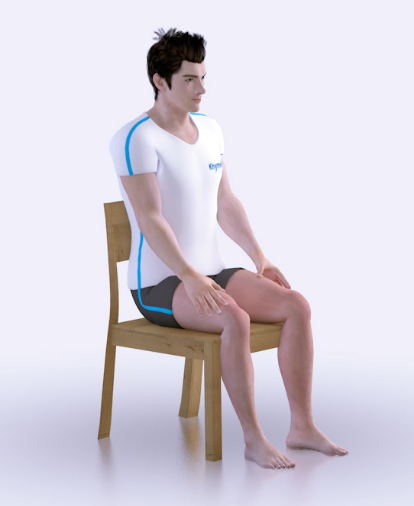 |
| Counterclockwise Scapular Circling | While seated, rotate your shoulder blades to perform large, slow, anti-clockwise circles as shown in the video | Keep your back straight | 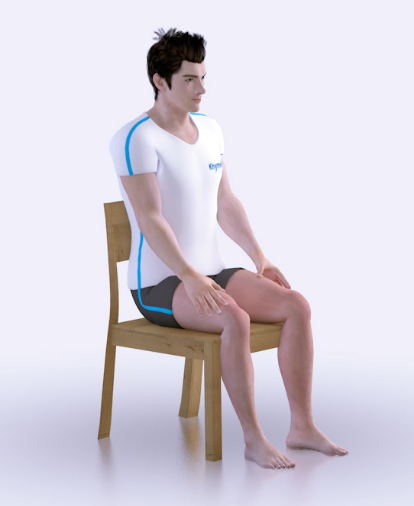 |
| Forward Humeral Circling | While seated, circle the arms wide and slowly forward | Keep your back straight | 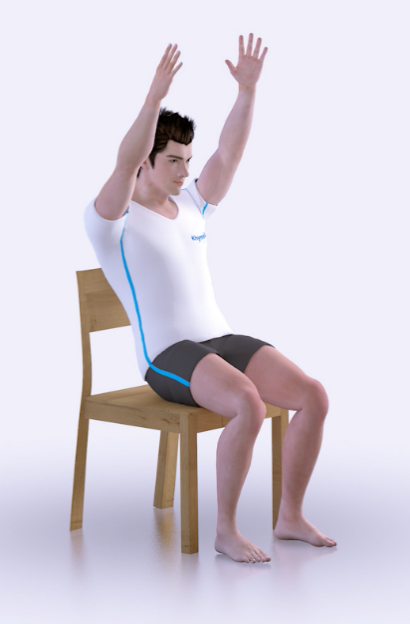 |
| Backward Humeral Circling | While seated, circle the arms wide and slowly backwards | Keep your back straight | 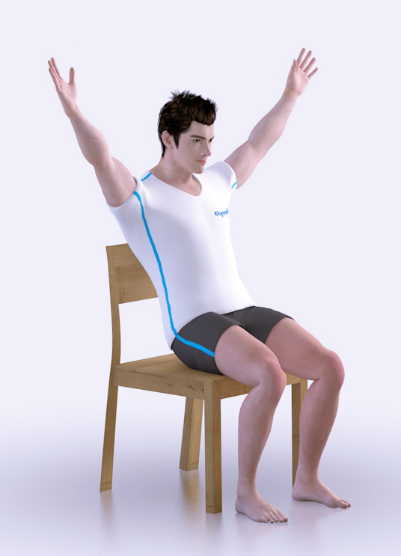 |
| Clockwise Cervical Circling | While sitting, perform wide and slow cervical circles in a clockwise direction | Keep your back straight Adjust the width according to the capacity | 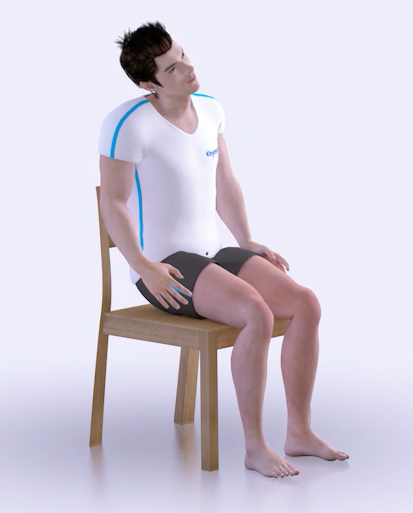 |
| Counterclockwise Cervical Circling | While sitting, perform wide and slow cervical circles in a counterclockwise direction | Keep your back straight Adjust the width according to the capacity | 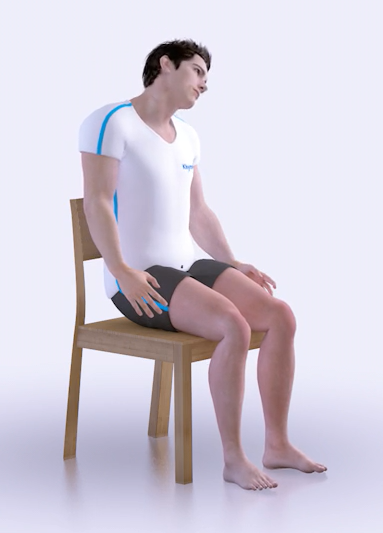 |
| Hip Flexion | Lie on your back, slowly flex your hip upward while keeping the knee straight | Keep your back straight Keep the knee straight | 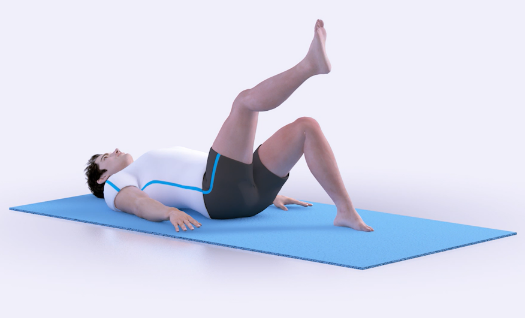 |
| Bridge | Lie on your back, lift your pelvis upward while maintaining the position for a few seconds as shown in the video | Keep your back straight Keep your arms crossed across your chest | 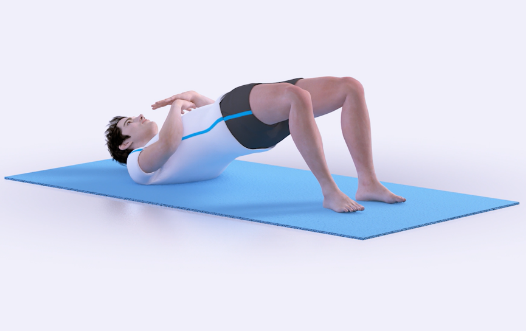 |
| Inverted Crunch | Lie on your back, bring your legs towards your chest while keeping 90 degrees between your thighs and back as shown in the video. Maintain the position for a few seconds | Maintain 90 degrees between the back and thighs | 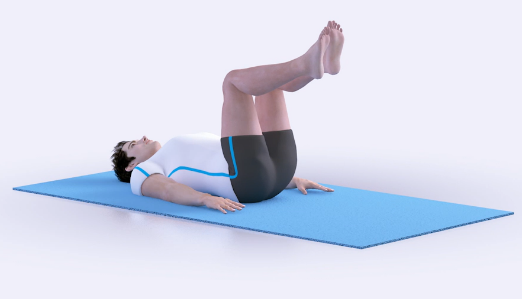 |
| Bird Dog (right) | Position yourself in a quadrupedal position and alternately raise an arm and leg opposite each other upwards. Repeat alternating positioning as shown in the video | Raise opposite leg and arm Alternate with each repetition | 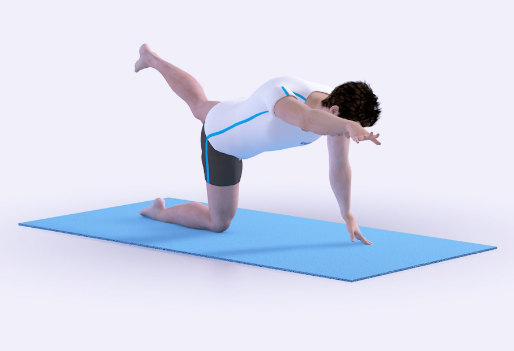 |
| Bird Dog (left) | Position yourself in a quadrupedal position and alternately raise an arm and leg opposite each other upwards. Repeat alternating positioning as shown in the video | Raise opposite leg and arm Alternate with each repetition | 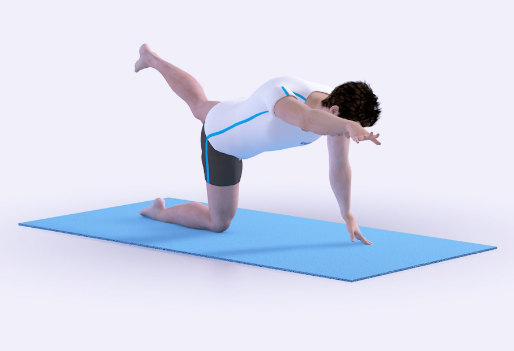 |
| Lateral Plank (right) | Position yourself in lateral decubitus resting on the forearm and knees. Lift your pelvis up and hold the position for a few seconds as shown in the video | Keep your legs aligned with your back | 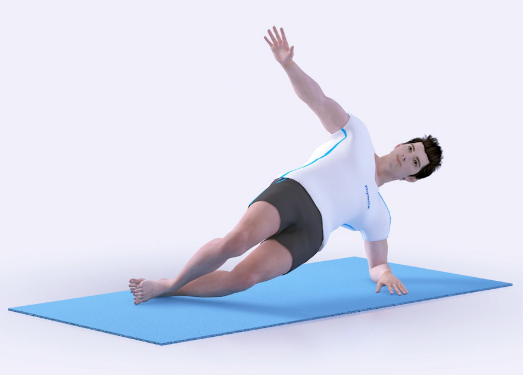 |
| Lateral Plank (left) | Position yourself in lateral decubitus resting on the forearm and knees. Lift your pelvis up and hold the position for a few seconds as shown in the video | Keep your legs aligned with your back | 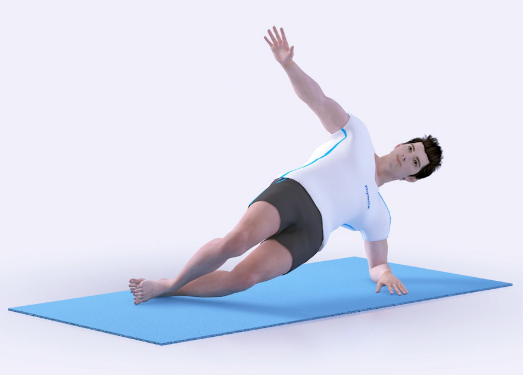 |
| Humerus Flexion | While seated, grab a stick and bring it up while keeping your elbows straight as shown in the video | Keep your back straight Possible to increase the load | 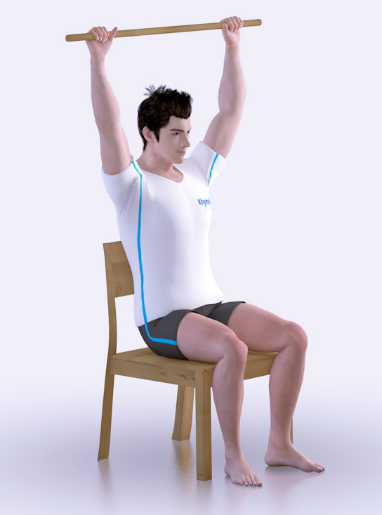 |
| Superior Reaching | While seated, grab a stick and push it up from the chest as shown in the video | Possible to increase the load | 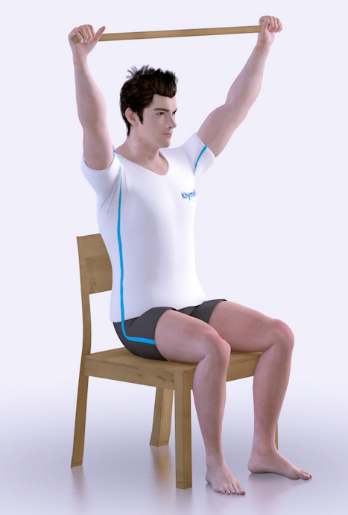 |
| Trunk Rotation 2 | While seated, grab a stick and bring it forwards while keeping your elbows straight. Once this position is reached, rotate the back slowly and broadly to the right and left trying to look backwards even with the gaze | Possible to increase the load | 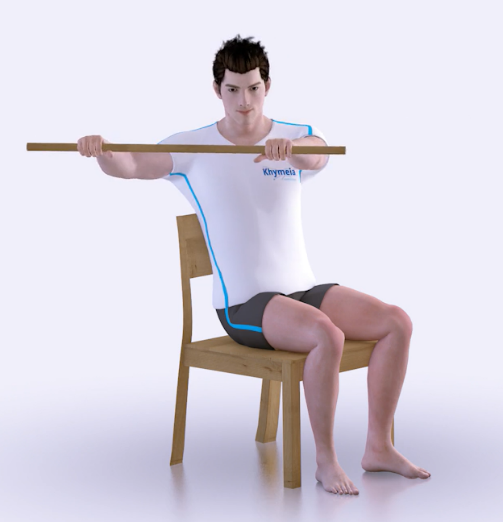 |
| Scapular Elevation | Standing up, grab a stick from the back and try to lift it as high as possible by sliding it down your back as shown in the video | Keep your back and head straight Possible to increase the load | 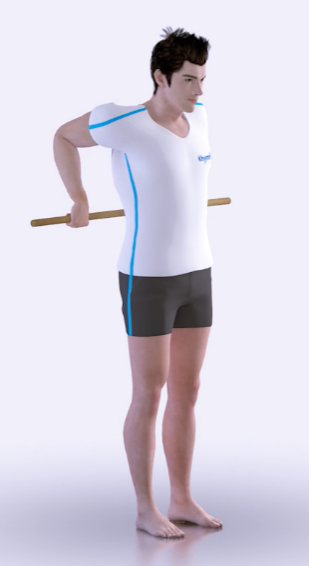 |
| Humerus Extension | Standing, grab a stick from the back and try to extend the arms back without flexing the elbows as shown in the video | Possible to increase the load | 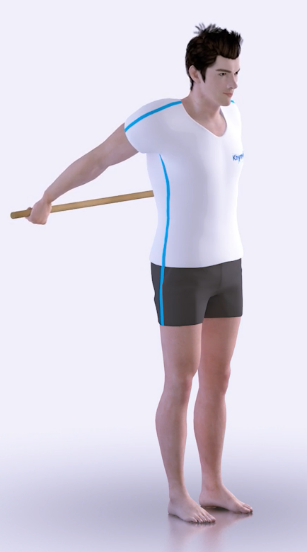 |
| Marching | March in place by lifting your knees up alternately as shown in the video | If necessary, support yourself on a chair or table to maintain balance | 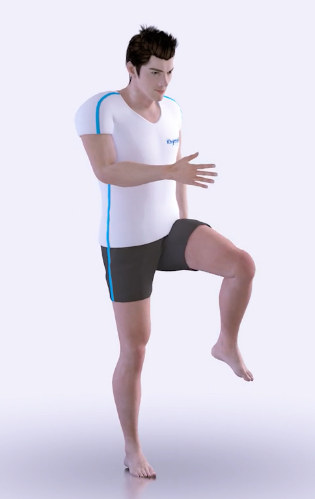 |
| Push-ups with tiptoes | Push on your toes trying to extend upward as shown in the video | If necessary, support yourself on a chair or table to maintain balance | 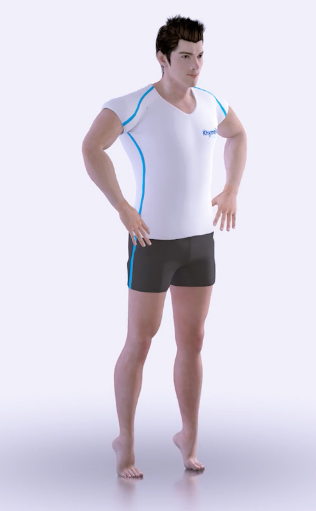 |
| Squat | Flex your knees and bend down slightly while keeping your back straight as shown in the video | If necessary, support yourself on a chair or table to maintain balance Keep your back straight | 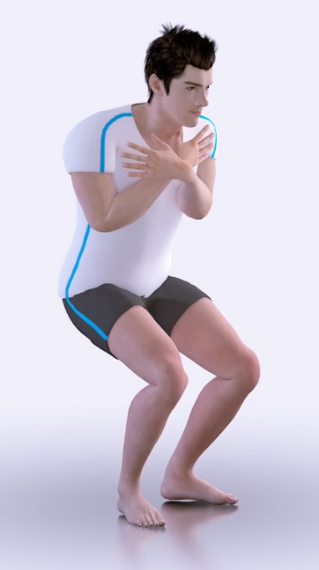 |
| Right Front Lunges | Perform a front lunge by bending the legs as shown in the video | If necessary, support yourself laterally to a chair or table to maintain balance | 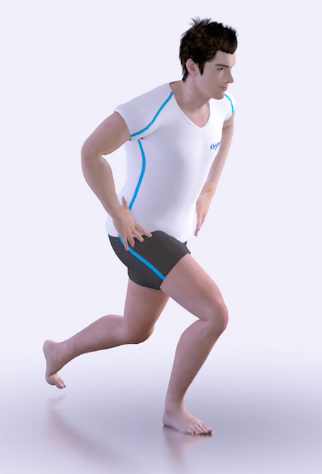 |
| Left Front Lunges | Perform a front lunge by bending the legs as shown in the video | If necessary, support yourself laterally to a chair or table to maintain balance | 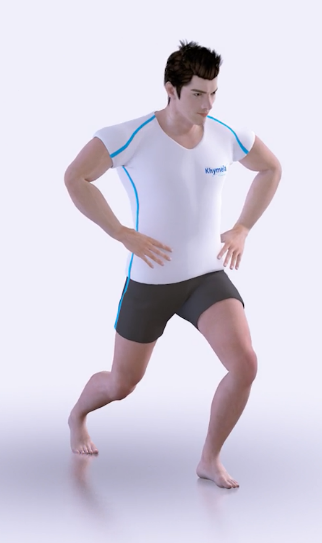 |
| Left Back Lunges | Perform a back lunge by bending the legs as shown in the video | If necessary, support yourself laterally to a chair or table to maintain balance | 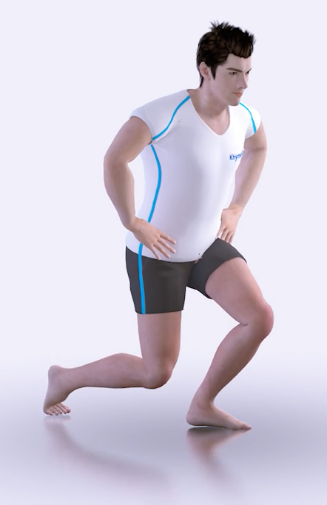 |
| Right Back Lunges | Perform a back lunge by bending the legs as shown in the video | If necessary, support yourself laterally to a chair or table to maintain balance | 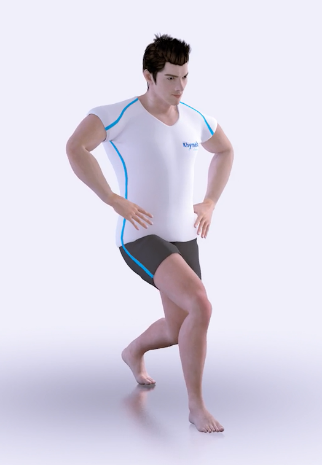 |
| Heels Walking | Walk on the heels of your feet for about 3-5 meters as shown in the video. Forward on the heels, go back on the heels | Perform the exercise with shoes | 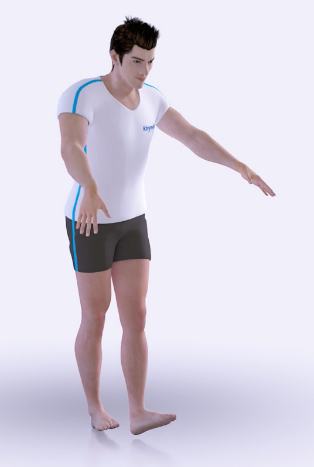 |
| Tiptoe Walking | Walk on your toes for about 3-5 meters as shown in the video. Forward on the tips, go back on the tips | Perform the exercise with shoes | 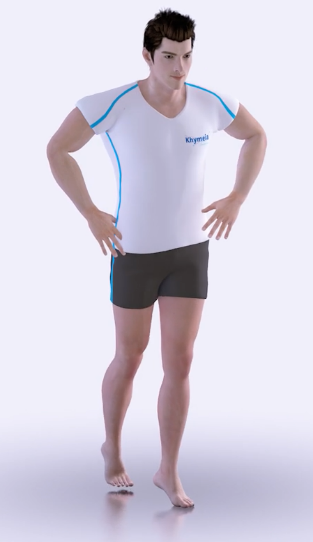 |
| Lateral Walking | Walk sideways for about 3-5 meters as shown in the video | Perform the exercise with shoes | 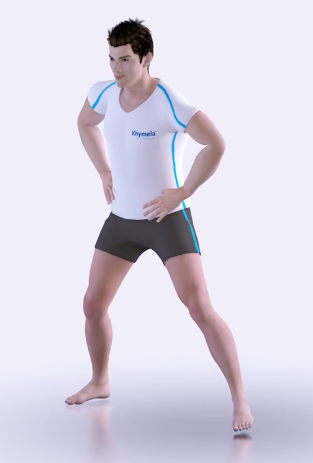 |

Supplementary Data: List of therapeutic exercises
