# Supplementary material for: Mobile application tool for remote rehabilitation after discharge from coronavirus disease‐19 rehabilitation unit
Source: Healthc Technol Lett. 2022 Aug 8;9(4-5):70–6. doi: 10.1049/htl2.12033 (PMC9535743; doi:10.1049/htl2.12033)
Supplement: Supplementary file 2 — Supporting Information [file HTL2-9-70-s001.docx]

| Subjects | Gender  (Male/Female) | Age  (years) | Weight  (kg) | Height  (m) | BMI  (kg/m^2^) | Education  (years) |
| --- | --- | --- | --- | --- | --- | --- |
| S_001_APP | M | 37 | 65 | 1.70 | 22.49 | 11 |
| S_002_APP | F | 54 | 58 | 1.66 | 21.04 | 16 |
| S_003_APP | M | 41 | 91 | 1.89 | 25.47 | 8 |
| S_004_APP | M | 55 | 59 | 1.70 | 20.41 | 18 |
| S_005_APP | F | 54 | 58 | 1.66 | 21.04 | 16 |
| S_006_APP | F | 70 | 60 | 1.65 | 22.03 | 11 |
| S_007_APP | M | 63 | 94 | 1.80 | 29.01 | 13 |
| S_008_APP | F | 56 | 70 | 1.67 | 25.09 | 8 |
| S_009_APP | M | 67 | 69 | 1.81 | 21.06 | 16 |
| S_010_APP | F | 83 | 71 | 1.60 | 27.73 | 5 |
| S_011_APP | F | 63 | 62 | 1.60 | 24.21 | 13 |
| S_012_APP | M | 71 | 83 | 1.67 | 29.76 | 13 |
| S_013_APP | F | 67 | 75 | 1.72 | 25.35 | 5 |
| S_014_APP | M | 50 | 76 | 1.65 | 27.91 | 13 |
| S_015_APP | M | 52 | 84 | 1.75 | 27.42 | 13 |
| S_016_APP | M | 73 | 76 | 1.75 | 24.81 | 13 |
| S_017_APP | M | 43 | 81 | 1.80 | 25.00 | 13 |
| S_018_APP | M | 75 | 101 | 1.80 | 31.17 | 5 |
| S_019_APP | M | 55 | 84 | 1.84 | 24.81 | 15 |
| S_020_APP | M | 76 | 64 | 1.70 | 22.14 | 11 |
| S_021_APP | M | 63 | 73 | 1.71 | 24.96 | 11 |
| S_022_APP | M | 75 | 64 | 1.70 | 22.14 | 8 |
| S_023_APP | M | 59 | 91 | 1.87 | 26.02 | 18 |
| S_024_APP | M | 61 | 90 | 1.80 | 27.77 | 20 |
| S_025_APP | M | 58 | 82 | 1.75 | 26.77 | 11 |
| S_026_APP | F | 48 | 69 | 1.60 | 26.95 | 10 |
| S_027_APP | M | 57 | 69 | 1.70 | 23.87 | 16 |
| S_028_APP | M | 61 | 80 | 1.70 | 27.68 | 8 |
| S_029_APP | M | 61 | 85 | 1.70 | 29.41 | 18 |
| S_030_APP | M | 62 | 74 | 1.70 | 25.60 | 24 |
| Mean (SD) | 8F / 22M | 60.33 (10.81) | 75.26 (11.64) | 172.16 (7.67) | 25.30 (2.92) | 12.7 (4.5) |

Supplementary Material_2: Gender, age, weight, height, BMI index and education of the 30 post COVID-19 patients. M: male; F: female. SD: standard deviation.
